# Assessment for Sustainable Use of Quarry Fines as Pavement Construction Materials: Part II-Stabilization and Characterization of Quarry Fine Materials

**DOI:** 10.3390/ma12152450

**Published:** 2019-08-01

**Authors:** Yinning Zhang, Leena Katariina Korkiala-Tanttu, Mari Borén

**Affiliations:** 1 Department of Civil Engineering, Aalto University, 00076 Aalto, Finland; 2 Destia Oy, Neilikkatie 17, 01301 Vantaa, Finland

**Keywords:** sustainable, secondary materials, quarry fines, freezing-thawing

## Abstract

A secondary by-product, quarry fines, has previously been investigated for applications in high volume as pavement construction materials. Results from a series of laboratory tests suggest qualified basic properties except for the possibility of frost susceptibility for the virgin quarry fines. In Part II of the research, stabilized quarry fine specimens were prepared and investigated in view of the mechanical behavior, and the durability represented by susceptibility to freezing and thawing cycles. The unconfined compressive strength, which is also the commonly used strength indicator, was adopted to evaluate the validity of the stabilized quarry fines as pavement construction materials. The laboratory-determined parameters were then compared among specimens treated with different stabilizers and with the typical requirements for pavement base/subbase layers. The stabilized quarry fines can be qualified for applications in pavement base, subbase and filter layer depending on the types of stabilizers used and degrees of compaction achieved.

## 1. Background

Quarry fine is a secondary by-product produced from the processing of rocks or aggregates yet of great material value for many civil engineering purposes. A huge amount of quarry fine materials is produced every year as a by-product of processing aggregates. Being an under-utilized material, the majority of it is just stored. At present, the most common application area of quarry fines in Finland is to use it as the uppermost unbound layer of yards, fields and low-volume streets [1]. Some research has also found that quarry fines could be used as an alternative to Portland cement for concrete [2], a stabilizer/additive to improve soil properties [2,3,4,5,6], and a substitute to sands/filler in concrete [7,8,9,10]. However, compared with the under-utilized portion, the quarry fine that has found its application in practice is still far from adequate. Unfortunately, these “leftover” quarry fine materials, if not appropriately treated, will lead to environmental and economic problems in society since the fine fraction of quarry fines will be easily dispersed by gravity, water and wind. Moreover, in the light of resource depletion and the promotion of a carbon-neutral society, wise utilization of existing resources is of vital importance. Under this circumstance, an effective way to utilize the quarry fine material in large volume is needed, and research activities regarding the characterization of this material to validate the promising applications are necessary.

As verified and stated in the previous research published earlier [11], basic properties of the unbound quarry fine materials have been investigated by a series of laboratory tests and analysis. Based on the particle distribution information obtained from both sieving and hydrometer methods, as well as the frost heave test results, it was found that the unbound quarry fines of 0–4 mm are prone to be frost-susceptible. The mechanical properties of quarry fine materials alone do not satisfy most of the requirements for a base or subbase as provided by many agencies. Thus, the stabilization of quarry fines to improve its properties for pavement base/subbase applications is potentially an effective way of utilizing it in higher volumes. The main motivations of the design, improvement and application of stabilized quarry fines as base/subbase material are listed as follows. 

(1) Conventional road construction materials of good quality, the majority as natural resources, are becoming scare due to the booming population, urbanization and industrial developments in the past few decades. Demanding sustainability and environmentally-friendly construction activities has become essential to all the countries encountering this problem. Numerous environmental constrains have been implemented by many countries, such as the EU Landfill Directive. 

(2) An increasing amount of industrial by-products have so far been stored, leading to environmental problems and waste of resources. As this problem has become more widely recognized, there have been increasing efforts to reuse industrial by-products in infrastructure constructions. Often these by-products have been used as stabilizers for treating weak soils. Several existing researches have demonstrated very promising results from utilizing industrial by-products to stabilize and improve the properties of various soil types. Results have shown that not only material strength, but also freeze-thaw performance, and compaction can be enhanced by applications of by-product binders. [12,13,14]. 

(3) There is a need for improving the long-term performance and increasing the lifespan of current infrastructure systems to achieve sustainable development. Therefore, higher requirements for material strength, stability and durability are necessary, and the application of stabilized base/subbase to achieve better performance is welcomed. Numerous types of recycled materials have been investigated for their effectiveness in cementation and ground improvement in previous research, including but not limited to Portland cement (PC), ground granulated blast furnace slag (GGBS), fly ash, recycled gypsum, cement kiln dust (CKD), bentonite and so forth [15]. All the existing experience in different types of stabilizers has been used as instructive information for stabilizing quarry fines as a pavement construction material in this study.

The aim of this follow-up study is to compare different binder materials to widen the usability of quarry fines, to define the mechanical properties of the stabilized samples, to assess their durability in laboratory, and to access their suitability as a pavement construction material. The tested mechanical property of the stabilized quarry fines was Unconfined Compressive Strength (UCS), whereas the durability of the stabilized quarry fines was further verified by a freeze-thaw test and capillary rise test. Last but not least, the feasibility of the quarry fine materials for pavement construction in the base, subbase or filter layer has been discussed. This study concentrates mainly on the mechanical and durability properties, not on the environmental (leaching or emission) issues.

## 2. Virgin Quarry Fine Materials

In the previous study [11], virgin quarry fine materials of 0 to 4 mm grain size, produced at the quarry in Koskenkylä by Destia Oy, were collected and investigated through a series of laboratory tests. The host rock of quarry is very high quality rock with extremely good abrasion value [16], which is only about 1% of what Finnish host rock material fulfills. Based on the results of previous research, the unbound quarry fine materials are shown to be good-quality and well-graded aggregates with both fine- and dense-graded characteristics. It has shown self-draining properties, but still a maximum dry density can be achieved, even though the influence of water content on dry density is low. The coefficient of permeability also complies with the conventional values in pavement base and subbase layers. In general, it is a promising material to be utilized for pavement constructions with regard to gradation, compactability and permeability. However, both the gradation information and frost heave test results have shown frost-susceptibility of the virgin quarry fines. 

To improve the performances of quarry fines, several types of stabilizers were used in this study. Further laboratory tests were performed to characterize the stabilized quarry fine materials and to access its feasibility in pavement constructions. Comparison analysis between cement-stabilized quarry fines (as a reference material) and quarry fines stabilized with other binders gives good evidence to show how effective different types of stabilizers are and to which part of the pavement the stabilized materials can be applied.

## 3. Characterization of Stabilized Quarry Fines As Construction Material for Pavements 

The main issue in the stabilization is to determine what type of stabilizer would be effective for better performance, cost-effectiveness and environmental friendliness. In this regard, the cement, which is one of the most commonly used and well established stabilizers for granular materials around the world, was selected as a reference material. It provided a baseline for comparisons with other types of by-product stabilizers: Ecolan and fly-ash stabilizers. These two types of potential stabilizers were proven to be effective as stabilizers, and their detailed components as well as the adopted ratios to quarry fines are introduced in the following sections. 

### 3.1. Specimen Preparation 

The appropriate dosage or proportion of each ingredient of stabilized material is the key factor to obtain better performance. The determination of mixture proportions requires a large amount of laboratory testing including aggregate gradation tests, reference density tests to determine the relationship between water content and dry density, selection of the relevant water content, and possibly activator content for each stabilizer. Moreover, the situation is even more complicated when it comes to the stabilized materials due to potential hydraulic reactions between the stabilizer and water. In this study, the mix proportions were selected either based on earlier laboratory and field test results, empirical knowledge, or suggestions from experienced agencies. The following section describes how the mixture design was determined for laboratory experiments.

- Cement type and content: For cement-stabilized quarry fines, quick cement by Finnsementti (pikasementti, Finnsementti Oy, Parainen, Finland) [17] was chosen because it is the purest calcium carbonate of current cement types, and many older researches have been made with relatively pure cement. Cement content was selected based on Finnish guidelines of overlay structures [18]. According to the guidelines, the typical cement content for stabilized soil is 2.5% to 5.0%. Considering the general experience that lower cement content is beneficial to the long-term performance of a stabilized base [19], as well as that for weather resistance the cement content should be more than 4.5% [18], the final two levels of cement content are determined to be 2.5% and 4.5% for quarry fine stabilization.
- Ecolan stabilizer contents: Ecolan stabilizer (Kolliantal och-slag, Renotech Oy, Turku, Finland) composed of coal ash, wood biomass, lime and cement, was also selected. The contents adopted for this stabilizer were selected to be 4.5% and 6.5% with suggestions from the earlier tests [20].
- Fly ash-based stabilizer content: Fly ash-based stabilizer is composed of 100% coal combustion residuals (CCRs) with a major component of fly ash. The stabilizer content was selected based on existing experience of the typical fly ash content as suggested for a stabilized base course by FHWA (federal highway administration) [21,22]. It suggests that a content of 12% to 30% of fly ash, with an activator of lime or cement adopted together, should be added to stabilize the pavement base course. Therefore, considering economy and environmental efficiency, two levels of low fly ash-based stabilizer content, 12% and 15%, were selected. Cement was also used as an activator in the fly ash-based mixture because the further pozzolanic reactions require an alkali condition to form cementitious Calcium-Silicate-Hydrate (C-S-H) gels and to stabilize the quarry fines. A cement content of 1% was adopted as an activator in the fly ash-stabilized quarry fines according to the FHWA recommended activator content of 0.5% to 1.5% for Portland cement [21].
- Optimal water content: The optimal water content of the unbound quarry fines was determined according to European standard in previous research [11]. The as-determined optimal water content of 9.26% was used as reference to select water content for adequate compaction.
- Water to cement ratio: Water-to-cement ratio was selected for effective hydraulic reactions when the quarry fines were stabilized with different stabilizers. According to recommendations by American Concrete Institute (ACI) [21] for the fly ash concrete, two levels within the typical range of 0.4 and 0.6 were adopted for stabilized quarry fines.

Following the designed ingredient proportions, raw materials of designed dosages were mixed in an electrical mixer thoroughly to get a batch for triplicated specimens. Each stabilized quarry fine specimen was fabricated by compacting a certain mass of material into a 50-mm-diameter mold in three layers until a final height of 100 mm and a degree of compaction of 85%, was reached. The compaction energy was kept the same for the three layers of triplicated specimens by keeping the same blow number and dropping from the same height, which also ensures a homogeneous specimen as much as possible. 

Since the 85% degree of compaction is the highest level that can be achieved by manual compaction, the specimens of 50 mm diameter were only used to determine the best mixture design. To achieve a higher degree of compaction, more specimens were prepared using the gyratory compactor with the best mixture design to evaluate performances of stabilized quarry fines.

### 3.2. Capillary Rise

Capillary actions driven by matric suction will pull water upwards to the frozen zone, whereas hydraulic conductivity will allow quantities of water to be transferred to the frozen zone. As a result, ice lenses are able to form continuously in the material and problems related with frost heave, and freezing-thawing can occur. In Part 1 [11], virgin quarry fines of grain size 0–4 mm have been proven to be frost susceptible based on laboratory-determined indicators: segregation potential. The effectiveness of applying stabilization techniques were evaluated by capillary rise test according to the European standard SFS-EN 13057: Products and systems for the protection and repair of concrete structures. Test methods. Determination of resistance of capillary absorption [23]. 

**Figure 1:**
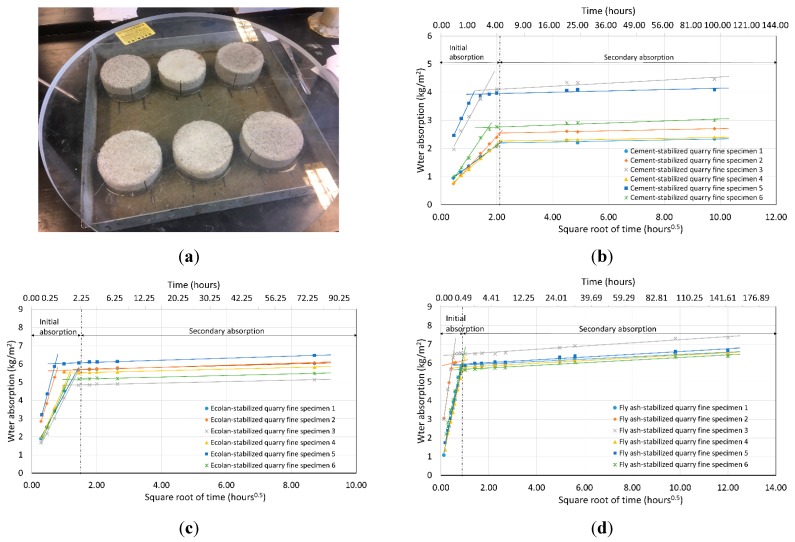
Capillary rise test: (**a**) specimens in one group; (**b**) water absorption of cement-stabilized quarry fines; (**c**) water absorption of Ecolan-stabilized quarry fines; (**d**) water absorption of fly ash-stabilized quarry fines.

There are different analyzing methods to obtain the absorption coefficient, including a 30 min method, one tangent method, and two tangent method [24]. The main difference of these methods is the way of selecting effective data points for the coefficient calculation. In this study, it was found that different methods have very limited influence on the final results, and one tangent method was used to determine the indicator “absorption coefficient” by following the European standard. The absorption coefficient was defined as the gradient of the prediction line between water uptake per unit area and square root of time (Figure 1). All the groups showed flat end portions, which indicated full saturation before the end of test, thus only the initial linear part of the graph has been used for gradient determination in this study.

The results show that the capillary sorption coefficients of stabilized quarry fine specimens are comparable to but still higher than the coefficients of concrete in the literature, if the same test method (EN 13057) is adopted. The average rate of water absorption for concrete cross-beam with silane of 20-year service life is from 0.247 to 1.033 mg/cm^2^ s^1/2^ [25]. The reported coefficients for hardened concrete containing recycled aggregate, air-entraining agent, cement, fly ash, and superplasticizer of varied proportions were from 0.746 to 1.549 mg/cm^2^ s^1/2^ (5.56 to 24.0 cm^2^/s) [26]. Similar ranges can be found in other studies where the coefficient is from 0.68 to 2.21 mg/cm^2^ s^1/2^ [27]. On the other hand, the coefficients for quick cement-stabilized quarry fine specimens were 1.24 to 3.56 mg/cm^2^ s^1/2^, and for quarry fine specimens stabilized with Ecolan or fly ash-based stabilizer, higher values from 5.85 to 11.36 mg/cm^2^ s^1/2^, and 9.27 to 14.41 mg/cm^2^ s^1/2^ were obtained. If compared with mixtures of river sand and natural hydraulic lime, similar water absorption coefficients between 7.9 and 19.2 mg/cm^2^ s^1/2^ have been published [24]. This coefficient for the building material was determined by European standard EN 1925 where a similar test method was used [28].

It can also be seen from Figure 1 that cement-stabilized quarry fines need a longer time to reach the status of full saturation compared with Ecolan-stabilized and fly ash-stabilized quarry fines, indicating that the Ecolan-stabilized and fly ash-stabilized quarry fines are more prone to absorb water and subject to freeze–thaw cycles. This finding can be further verified by the results of the freeze–thaw test presented in the following sections.

### 3.3. Unconfined Compressive Strength

#### 3.3.1. Determination of the Mix Design

Unconfined compressive test is one of the methods that is normally adopted to determine the strength of bound materials. Unconfined Compressive Strength (UCS) is a typical strength parameter used by not only researchers but also industries in civil engineering to verify if the mixture is suitable for applications with satisfactory performance. There are also empirical relationships developed to predict other material properties such as resilient modulus from the unconfined compressive strength. In pavement engineering, many research reports, specifications and standards have suggested the minimum UCS of stabilized material for base and subbase layers of pavement. In this consideration, the UCS has been selected in this study as the indicator to determine the best mix design for stabilizing quarry fines. Table 1 lists some of the requirements as provided by different agencies.

The standard test method Geotechnical investigation and testing. Laboratory testing of soil. Part 7: Unconfined compression test (ISO 17892-7:2017) was followed to determine the unconfined compressive strength. Stabilized quarry fine specimens of 50 mm diameter and 100 mm height were compressed at a strain rate of 1.0 mm per minute (between 1% and 2% of the specimen height per minute) while the load, displacement and elapsed time were recorded for further data processing. At a given mix design, triplicated quarry fine specimens were tested successively and the average was adopted in data analysis. Table 2 lists all the mixture designs of the specimens and the highest compressive strength together with the best mixture option that has been achieved. 

The maximum compressive strength of quarry fine specimens stabilized with quick cement at 85% degree of compaction reached around 2.5 MPa for a 28-day curing period in this study, compared to a lowest strength of 0.703 MPa that is only 28.1% of the maximum strength. This result indicates the profound influences of mixture design, or the stabilizer and water content, and associated compaction energy on the final strength of stabilized quarry fine materials. It is found in this study that a lower water-to-binder ratio and higher cement content added to the quarry fine specimen brings higher compressive strength of cement-stabilized quarry fine mixture. The same trends can be found for quarry fines stabilized with fly ash and an activator of cement. Nevertheless, different situations can be observed for other types of stabilizers. When the Ecolan stabilizer is applied, a higher water-to-stabilizer ratio and stabilizer content are needed to get higher compressive strength. It is also found that the fly ash itself has shown little improvement to the strength of quarry fines, but with a small amount of activator (e.g., cement) added, higher strength can be achieved. Generally, quarry fine specimens stabilized with quick cement have the highest UCS, whereas those stabilized with other binders consisting of coal ash with the same or even higher stabilizer content are less strong. 

**Figure 2:**
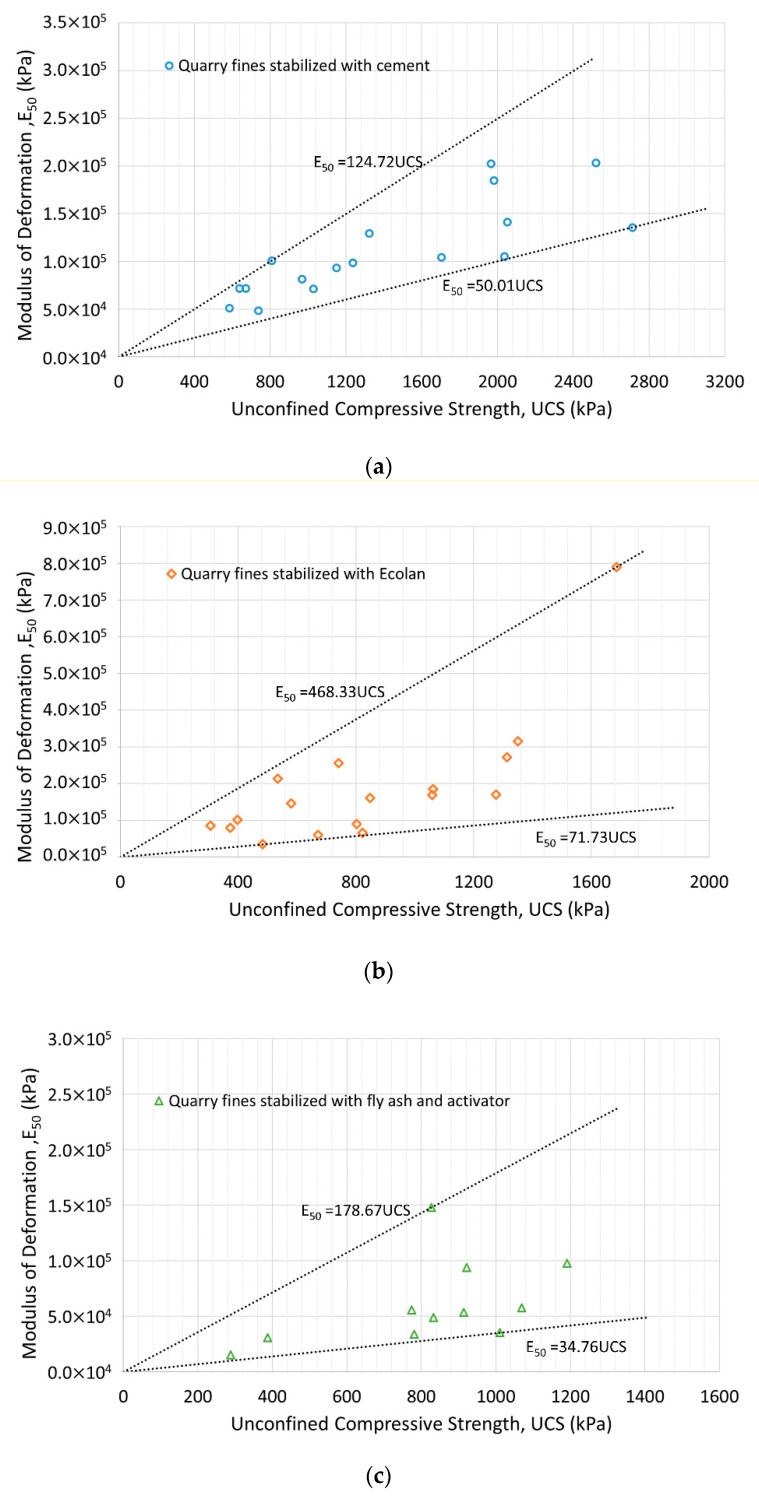
Modulus of deformation versus unconfined compressive strength of stabilized quarry fines: (**a**) Quarry fines stabilized with cement; (**b**) Quarry fines stabilized with Ecolan; (**c**) Quarry fines stabilized with fly ash and activator.

The undrained shear strength predicted from the UCS, as well as the secant modulus or modulus of deformation E_50_, which was obtained from the data corresponding to half the maximum UCS [35,36], are also listed in Table 2. Figure 2 presents the relationship between the UCS and modulus of deformation from the laboratory test data for stabilized quarry fine specimens of different stabilizer content, water content and compaction energy. It is found that the modulus of deformation E_50_ of cement-stabilized quarry fines can be predicted as having an approximate value of 5 to 125 × UCS, even though the strength and modulus of cemented quarry fines are much higher than that of the cemented clay. For quarry fines stabilized with Ecolan and fly ash-based stabilizers (with activator), the coefficients are of 72 to 468 × UCS and of 35 to 179 × UCS respectively. The multiplier coefficients of Ecolan-stabilized quarry fines are higher than the typical coefficients of undisturbed cemented clays from 50 to 300 [37], while the fly ash-stabilized quarry fines have similar coefficients to undisturbed cemented clays.

On the other hand, it can be seen from Table 2 that part of the compressive strength satisfies the requirements provided by several agencies as listed in Table 1. However, it is not convincing to qualify the best mixture for applications in pavement subbase and even base layers, considering that the quarry fines stabilized by Ecolan and fly ash-based stabilizers are around or below the lowest requirement, and much higher thresholds are required by the other agencies. Moreover, to resist frost deteriorations in cold regions, a higher compressive strength should be satisfied. Therefore, the test results as presented previously are only utilized to determine the best mixture design and the appropriate curing conditions. Considering that the degree of compaction of these specimens is 85% but most of the agencies require a higher degree (e.g., >90% in Finnish guidelines) especially for pavement base/subbase layer, higher strength is necessary and achievable for the same mix design at a higher compaction energy.

#### 3.3.2. Influence of Degree of Compaction and Curing Days

**Figure 3:**
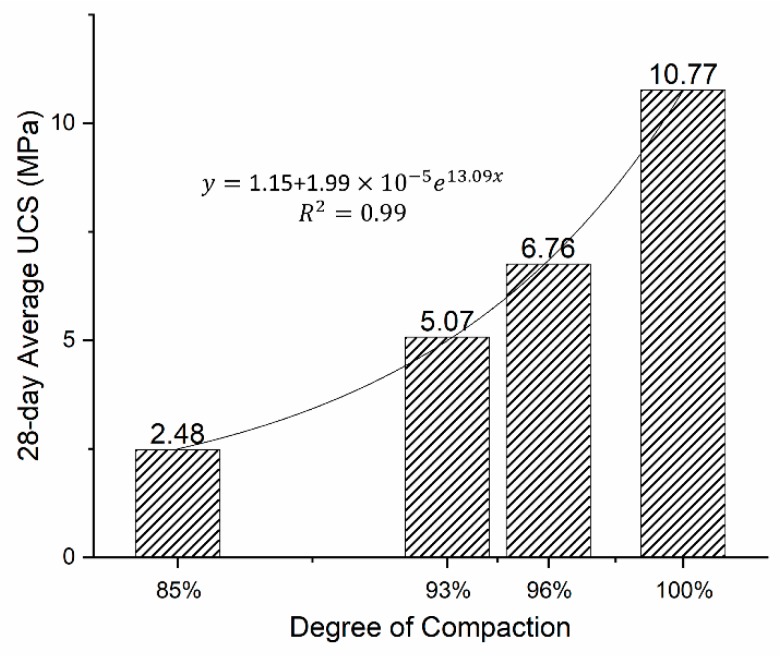
Effects of degree of compaction on the unconfined compressive strength.

Figure 3 shows that the degree of compaction has significant effects on the unconfined compressive strength for the cement-stabilized quarry fine specimens. The 100% degree of compaction does not imply a practical status of zero air voids, but is the highest degree of compaction that can be achieved by a gyratory compactor after 512 gyrations. It can be seen from the figure that the value of UCS has increased exponentially as the degree of compaction increased from 85% to 100% and that the UCS of stabilized quarry fine specimens of 96% degree compaction can adequately satisfy the requirements of pavement base/subbase provided by different agencies. It is therefore concluded that stabilized quarry fine materials can be qualified for pavement base/subbase applications in term of unconfined compressive strength, as long as appropriately designed and constructed. A least degree of compaction of 93% is recommended for cement-stabilized quarry fines with 0–4 mm grain size and 4.5% cement content for such purposes. For quarry fines stabilized by Ecolan, or fly ash and activator, at least a 93% or higher degree of compaction is necessary to validate their applications in base or subbase. Therefore, more stabilized quarry fine specimens with the best mixture design were prepared by gyratory compactor to achieve at least a 93% degree of compaction, and the following characterization of stabilized quarry fines is only based on these denser specimens.

**Figure 4:**
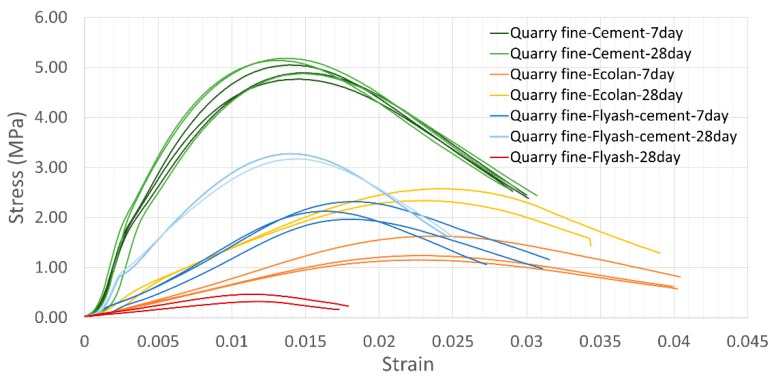
Effects of curing time on the unconfined compressive strength of stabilized quarry fines.

Other than degree of compaction, the duration of curing time also influences the strength of stabilized quarry fines. In this study, the curing condition of 100% humidity was applied for all the stabilized quarry fine specimens of all the types of stabilizers. Figure 4 displays the influence of curing time on the UCS of stabilized quarry fines.

**Figure 5:**
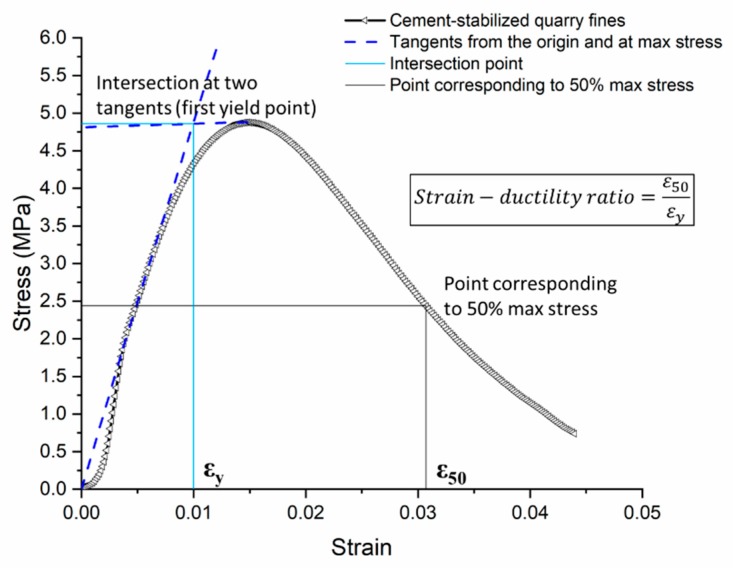
Strain–ductility ratio from stress–strain relationship.

From the relationships between stress and strain in Figure 4, it can be seen that the type of stabilizer and mix design have influenced the way of deformation and failure under the same loading conditions. Quarry fines stabilized with Ecolan stabilizer have higher strains at failure whereas the cement-stabilized quarry fines demonstrate the most similar stress–strain relationship/shape of curve for different curing times. To better present the effects of curing time, an indicator based on “deformation-ductility ratio”, which is originally developed for reinforced concrete, was developed and adopted. As shown in Figure 5, the strain–ductility ratio (SDR) is capable of considering the strains between the first yield point and the point where the strength is reduced to a certain percentage, serving as a good indicator for evaluating the ductility of the material. Table 3 lists the strain–ductility ratio (Equation (1)) of different stabilized quarry fines of 7-day and 28-day age, showing that cement-stabilized quarry fine is the most stable mixture regarding curing time. Ecolan-stabilized and fly ash-stabilized quarry fines all became more ductile and easier to yield, whereas the fly ash-stabilized quarry fines show considerable increase in ductility within curing time.

(1) $$Strain-ductility\;ratio=\frac{\varepsilon_{50}}{\varepsilon_{y}}$$

where $\varepsilon_{50}$ is the strain corresponding to 50% of the compressive strength after failure, and $\varepsilon_{y}$ is the strain at first yield point.

The change in strength and ductility is linked to the continuous reaction between water and the binders during the curing period. The changes in strength due to curing time also vary among different mixtures. It is interesting to see that a longer curing time with the presence of water does not necessarily have positive effects on the development of unconfined compressive strength for stabilized quarry fine materials. In fact, the compressive strengths of cement-stabilized quarry fines are hardly changed even undergoing a longer period of curing time from 7 days to 28 days, with an average of 4.91 MPa and 5.07 MPa respectively. It is possibly related with the fast strength development of quick cement as a stabilizer for quarry fines, so that a longer period of exposure to moisture can be harmful to the strength of cement-stabilized quarry fines. However, when Ecolan or fly ash plus cement was used as a stabilizer, the strength increased by 78.6% and 42.5% from 7 days to 28 days.

#### 3.3.3. Comparison with Requirements

**Figure 6:**
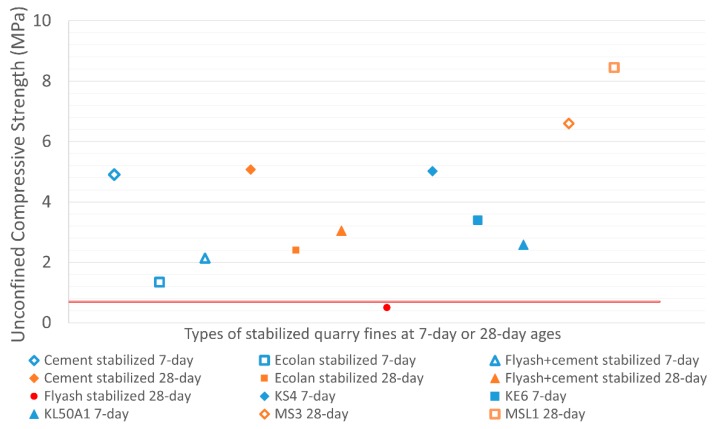
Average unconfined compressive strength of stabilized quarry fine specimens.

To validate the stabilized quarry fine materials for pavement constructions in terms of unconfined compressive strength, unconfined compressive strength tests were conducted only on stabilized specimens of a higher degree of compaction (93% and 96%). These specimens were prepared using the gyratory compaction method, following the most promising mixture design as obtained from the previously mentioned investigation. Typical strength limits for pavement base, subbase and subgrade as listed in Table 1 were used to assess the potential applications of the stabilized quarry fine materials. Figure 6 shows the average unconfined compressive strength of each mix based on triplicated specimens.

It should be noted that only 7- and 28-day ages were used in this research so that the stabilizations mainly based on hydraulic reactions are considered, rather than the slow-setting pozzolanic and carbonation reactions.

In fact, the lower limit of 0.7 MPa as plotted in Figure 6 is the minimum requirement of all the recommended lower limits by different agencies as listed in Table 1. It can be seen as the lowest limit that should be satisfied to qualify high-volume applications of stabilized quarry fine materials for pavement construction, in either base, subbase or subgrade. Fly ash itself has been proven to be not suitable to stabilize quarry fines because of its low strength. However, when a small amount of cement is used as an activator, the strength can be improved significantly and the stabilized quarry fines would have adequate strength (28-day, about 3 MPa) even for the base layer in medium to high volume roads according to Portland Cement Association. The other agency’s requirements also suggest its adequate strength for applications in subbase and subgrade. However, the quarry fines stabilized with Ecolan are more suitable for subbase and subgrade according to the agencies listed in Table 1. When quick cement is used as a stabilizer, stabilized quarry fines can be satisfactorily qualified by most of the agencies as base material in medium to high volume roads, in terms of unconfined compressive strength.

Results from previous research are also plotted in Figure 6 for comparison [20,38]. The specimens KS4, KE6 and KL50A1 are quarry fines (0–3 mm) stabilized with plus cement, Ecolan stabilizer and 50% fly ash with 1% cement as activator. The 7-day unconfined compressive strength of cement-stabilized specimens is quite similar, while for the other two types of stabilizers, the previous research obtained higher strengths mainly due to higher degree of compaction (DOC, 96%). If the gradation of aggregates was improved by adding more coarse fractions such as crushed rock of 0–20mm, the strength will be improved significantly as shown for specimens MS3 (5% plus cement) and MSL1 (2% plus cement + 6% fly ash).

#### 3.3.4. Failure Modes

**Figure 7:**
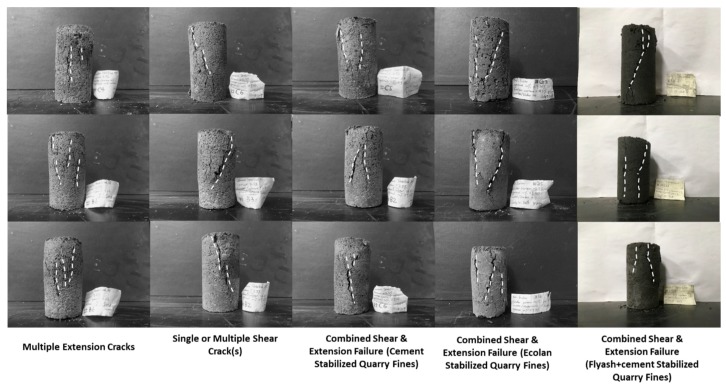
Cracking types (first two columns) and major failure mode (column three to five) of the stabilized quarry fine specimens in unconfined compressive test.

The major cracking types developed in the stabilized quarry fine specimens after unconfined compressive test are shown in Figure 7, where two types of cracks caused by extension and shear have been observed. However, in this study, the combinations of tensile splitting and inclined shear cracks are the main failure modes, and the barrel-shaped specimen after testing with cracks indicates semi-plastic characteristics of the stabilized quarry fine materials. Among all 18 quarry fine specimens stabilized with quick cement, 16 of them are observed to have combined extension and shear cracks as the failure mode, while the other two are found to have only extension cracks after compression. Therefore, combined extension and shear failures can be recognized as the main failure mode of cement-stabilized quarry fines under unconfined compression conditions.

The same main failure mode of combined extension and shear can also be observed for the Ecolan-stabilized and fly ash plus cement-stabilized quarry fine specimens. Two out of the total 18 Ecolan-stabilized specimens were found to have multiple extension cracks while the others all have combined extension and shear failures. For quarry fines stabilized with fly ash plus cement, two out of the total 12 specimens only have failure mode of multiple extension cracks and the rest all have combined cracks. There seems to be no significant difference in failure mode for the quarry fines stabilized by the three types of stabilizers as adopted in this study. 

### 3.4. Freeze–Thawing Properties

Ground freezing and thawing is a result of the annual cycle of temperatures, which is also a common situation encountered in Nordic countries. A recent study found that with global climate changes, the intensity of thaw weakening will be more serious [39], and the annual number of freeze–thaw cycles is increasing. As a candidate construction material, stabilized quarry fines should be capable to sustain in such severe environmental conditions. The resistance of the stabilized quarry fines to the cyclic action of freezing and thawing is critical to the performances of buildings and infrastructures in cold regions, and thus is estimated by freeze–thaw testing. The technical specification CEN/TS 13286-54 Test method for the determination of frost susceptibility. Resistance to freezing and thawing of hydraulically bound mixtures was followed to conduct the test on stabilized quarry fines [40].

In the first stage, cylindrical stabilized quarry fine specimens of 100 mm diameter and height were prepared using gyratory compactor ICT to achieve 96% degree of compaction. The specimens for freeze-thaw testing were fabricated according to the most promising mix designs to achieve the highest UCS as obtained from previous tests. For each type of stabilizer, six specimens were fabricated, kept in mould, and cured in a humidity room for 28 days. Then they were removed from the mould and divided into Set A and B with three specimens in each. As a result, the first stage was finished to allow adequate strength development for the specimens. The second stage consists of the application of freeze-thaw cycles and the strength test. Both of the two sets were placed in a humidity room to prevent the loss of moisture, and Set A was removed from the humidity room after 2 days and wrapped and placed in a low temperature cabinet for 10 freeze-thaw cycles, whereas Set B was kept in a humidity room until the conditioning of Set A was finished. Then all the specimens were used in an unconfined compressive strength test and the freeze–thaw retained strength ratio was calculated. 

**Figure 8:**
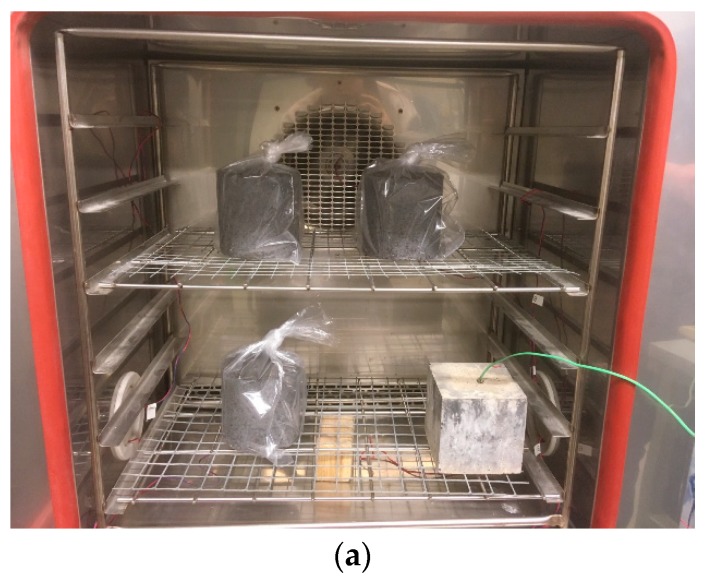

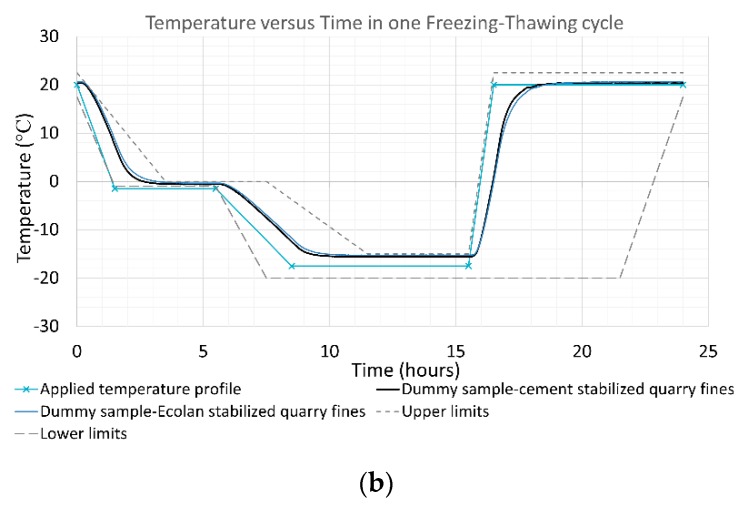
Freeze–thaw test: (**a**) Stabilized quarry fine specimens inside the chamber; (**b**) Temperature profile applied in freeze–thaw cycles.

As shown in Figure 8, one freeze-thaw cycle takes 24 hours to finish, and the controlled temperature inside the low temperature cabinet was within the limits as suggested by the specifications. The monitored temperature was measured inside a cubic cement concrete specimen placed inside the cabinet.

Based on the unconfined compressive strength of both Set A and B quarry fine specimens subjected to none or 10 freeze-thaw cycles, no distinguished differences were found between the two sets. However, still the average UCS of regular specimens is higher than that of conditioned specimens, and the strain corresponding to the UCS at failure has increased. Based on calculations, some indicators including the previously mentioned strain-ductility ratio, the freeze-thaw retained strength ratio is presented in Table 4. 

Where FTR is the freeze–thaw retained strength ratio $\frac{R_{A}}{R_{B}}$, and $R_{A}$ and $R_{B}$ are the mean value of strength for Set A and Set B, respectively.

Subjected to 10 freeze-thawing cycles, the specimens have been deteriorated in terms of reduced UCS and strain-ductility ratios. As shown in Table 4, given the same external loading conditions, the specimens have become less ductile after the cyclic freeze-thawing conditions, whereas the strains at first yield point and at failure all increase. This can be further interpreted as the materials subject to freeze-thawing cycles were deformed more before failure but became more brittle and easy to break after the peak stress. From the curves of cement-stabilized quarry fines, there are obvious relaxation stages after the beginning of the load for the conditioned specimens, contributing to the total strains at failure. On the other hand, based on the freeze-thaw retained strength ratio, it can be seen that the deteriorations caused by 10 freezing and thawing cycles for the cement-stabilized quarry fine specimens were not significant. This qualifies the cement-stabilized quarry fines as a pavement construction material for different layers. The deteriorations caused by freeze-thawing cycles for Ecolan-stabilized quarry fines were more serious, which resulted in a decrease in the UCS by about 20%. Yet, this amount of deterioration in the UCS is normal for stabilized granular materials such as lime-treated kaolinite [41,42] and fiber-reinforced clay [43,44]. However, improvement is still necessary to enhance the freeze-thawing resistance for quarry fines stabilized with fly ash, even though the ratio is right on the limit set by Finnish guidelines of 6.7 [18]. The fundamental mechanism behind such a difference in freeze-thawing resistance of quarry fines stabilized with different stabilizers stills need to be investigated from a finer-scale point of view. Related research and results will be introduced in the future.

## 4. Discussion on Potential Applications of Quarry Fine Materials

Existing research has already presented several applications of virgin quarry fine materials, including as a partial substitute to cement and as an additive to improve the properties of soils, whereas these applications are only in limited volumes. The virgin quarry fines of 0–4mm as adopted in this study have a similar particle size distribution to well-graded sand, and a coefficient of permeability around 5.75 × 10^−5^ m/s [11] indicates its potential material value in construction for civil engineering purposes. However, considering that the previous test results have also suggested possible frost-susceptive properties, stabilizers were applied to improve the properties of virgin quarry fines as a construction material. Unconfined compressive strengths of the stabilized quarry fine materials were investigated by laboratory tests, which further verified the potential of the stabilized quarry fines for civil engineering applications from the strength point of view.

Based on the typical strength and durability requirements for materials used in pavement structural layers, the stabilized quarry fine materials can qualify for high-volume civil engineering applications, as long as they are well designed and compacted to enough degrees. One of the most possible applications is in road construction. The stabilized quarry fines can be qualified for base, subbase, filter layers, and subgrade depending on the different stabilizers used and degrees of compaction achieved. Nevertheless, applications for pavement subbases are much more suggested. This is because higher quality material is better for base layers to ensure pavement performances in the long run, and unbound material is more favorable for the filter layers considering cost efficiency. The other possible applications include but are not limited to embankment building, landfill construction, and construction site leveling, where strength requirements are not high. 

However, when the strength requirements are high, it is still possible to utilize the stabilized quarry fine materials. It is well-established that particle size distribution of the aggregate has shown important effects on the unconfined compressive strength of mixtures. This has also been seen when comparing the results of this research with the previous ones. When coarse particles are added to quarry fines to obtain a good gradation curve and compaction, the strength can be improved significantly. Therefore, in situations where higher strength is required, either modifying the gradation of quarry fines by adding more coarse fractions or increasing the DOC level (if achievable in field) would be options. 

On the other hand, even though the resistance to freeze-thawing cycles of the stabilized quarry fines is acceptable based on laboratory results in this study, the detrimental effects of salt have been found somewhere else. In situations where freeze-thawing cycles are presented and deicing agent is applied, the resistance would be impaired especially for the cement-stabilized quarry fines. It is therefore suggested that for pavements where deicing agent is needed, a waterproof asphalt concrete surface layer would be helpful to reduce the salt infiltration and maintain the resistance of the underlying stabilized quarry fine layers to freeze-thawing cycles.

It should be noted that all these potential high-volume applications are based on the assumption that the quarry fine materials can satisfy all the environmental requirements set by the government. Also, there are still some uncertainties of the conclusions made in this study, mainly due to the limited number of specimens that has been tested and the possible variations of the material quality over different quarries. 

## 5. Conclusions

- It is found that the modulus of deformation E_50_ of cement or fly ash (with activator) stabilized quarry fines can be predicted with a similar multiplier coefficient to cemented clays. For quarry fines treated with Ecolan stabilizer, the multiplier coefficients are higher than the typical coefficients of undisturbed cemented clays. The differences in multiplier coefficients among mixtures are mainly due to different virgin material properties, water content and the type of stabilizer applied.
- The UCS of stabilized quarry fine specimens of 96% degree of compaction can adequately satisfy the requirements of pavement base/subbase provided by different agencies in terms of strength, as long as appropriately designed and constructed. A least 93% degree of compaction is recommended for cement-stabilized quarry fines with the proposed mixture design, to be qualified for applications in base layers. When Ecolan or flyash with cement is used as the stabilizer, a higher degree of compaction would work better.
- Higher cement content and a lower water-to-cement ratio will result in stiffer cement-stabilized quarry fine materials. A similar situation was also observed on quarry fine specimens stabilized by the fly ash and activator. The opposite situation was found for the Ecolan stabilizer where a higher stabilizer content and water-to-stabilizer ratio will lead to higher strength.
- Combined extension and shear failures can be recognized as the main failure mode of stabilized quarry fines under unconfined compression conditions.
- The specimens subjected to freeze-thawing cycles have been deteriorated in terms of reduced bearing capacity and the increased deformation given the same external loading conditions. The freeze-thaw retained strength ratio indicates that the deteriorations caused by 10 freeze-thaw cycles were very limited for cement-stabilized quarry fines which can be qualified as a pavement construction material. For the Ecolan or fly ash-stabilized (with activator) quarry fines, higher susceptibilities, yet within the requirements, were observed, indicating a need for improvements. The results of the capillary rise test also support this finding.
- When the strength requirements are high (such as for base layer), it is still possible to utilize the stabilized quarry fine materials by modifying the gradation or increasing the DOC level (if achievable in field).
- For pavements where a deicing agent is needed, a waterproof asphalt concrete surface layer is suggested to maintain the resistance of the underlying stabilized quarry fine layers to freeze-thawing cycles.

## 6. Future Research

In this research, varied stress-strain relationships under the same loading conditions for the quarry fine specimens stabilized with different stabilizers have been observed. In the future research, another mechanical test, such as Repeated Load Triaxle (RLT) test, may be conducted to further investigate its mechanical properties. Fundamental mechanisms lying behind this, as well as the different UCS values caused by different binding effects of various stabilizers, still need to be investigated. A future study based on more fundamental, fine-scaled investigation with Scanning Electron Microscopy (SEM) and Energy Dispersive Spectroscopy (EDS) techniques will be conducted to compare mineral compositions of quarry fines and host rock, and to explain the binding and freeze-thawing effects on quarry fines.

## Figures and Tables

**Table 1:** Unconfined compressive strength requirements for base/subbase/subgrade of pavements.

References	Minimum or Typical UCS Ranges (MPa)	Curing Period	Applications
Portland Cement Association [29]	2.068–5.516 (300–800 psi)	7 days	Medium- to high-volume roads for subbase/base
Mechanistic-Empirical Pavement Design Guide (MEPDG) [30]	1.72 (250 psi)	7 days for cement and 28 days for lime-fly ash or cement fly ash	for subbase, subgrade
	5.17 (750 psi)		for base
Austroads [31]	2.0	28 days curing and 4 h soaking prior to testing	bound pavement materials (stabilizer > 3%)
	1.0–2.0		subgrade, lightly-bound pavement materials (stabilizer < 3%)
Federal Highway Administration (FHWA) [32,33]	4.1	N/A	for stabilized drainable base in cold regions to resist frost deterioration
	0.7	7-day cure at 105 °F (40 °C)	subgrade lime/soil
	1.0	7-day cure at 105 °F (40 °C)	subgrade lime/fly ash/soil
	1.4	7-day cure	subgrade cement/soil, cement/fly ash/soil, or fly ash/soil
InfraRYL [34]	3.0–8.0	7-day cure	cement-stabilized
	5.0–13.0	28-day cure	cement-stabilized
	1.0–2.0	28-day cure	blast furnace slag stabilized

**Table 2:** Types of stabilized quarry fine specimens and their strengths.

**Quick Cement Stabilized Quarry Fine Specimen #**	**Stabilizer Content**	**Water to Binder Ratio**	** Unconfined Compressive Strength, kPa	**Undrained Shear Strength, kPa**	**Secant Modulus/Deformation Modulus E50, MPa**
A1–A3	4.5%	0.4	1741.2	870.59	172.08
A4–A6	4.5%	0.6	1924.4	962.22	115.10
B1–B3	2.5%	0.4	758.8	379.41	60.68
B4–B6	2.5%	0.6	703.5	351.77	82.63
C1–C3*	4.5%	0.0	2483.2	1241.59	196.64
C4–C6	2.5%	0.0	1128.0	563.98	87.92
**Ecolan Stabilized Quarry Fine Specimen #**	**Binder Content**	**Water to Binder Ratio**	** Unconfined Compressive Strength, kPa	**Undrained Shear Strength, kPa**	**Secant Modulus/Deformation Modulus E50, MPa**
E1–E3	4.5%	0.4	632.9	316.44	142.16
E4–E6	4.5%	0.6	713.2	356.59	95.85
F1–F3	6.5%	0.4	993.6	496.82	167.09
F4–F6*	6.5%	0.6	1316.7	658.35	225.64
G1–G3	4.5%	0.0	365.0	182.51	90.38
G4–G6	6.5%	0.0	981.7	490.83	273.83
**Fly ash + Cement Stabilized Quarry Fine Specimen #**	**Binder Content + Activator Content**	**Water to Binder Ratio**	** Unconfined Compressive Strength, kPa	**Undrained Shear Strength, kPa**	**Secant Modulus/Deformation Modulus E50, MPa**
H1–H3 *	12.0% + 1%	0.0	846.1	423.08	105.85
H4–H6	15.0% + 1%	0.0	571.3	285.67	42.44
H7–H9	12.0% + 1%	0.6	795.1	397.54	43.41
H10–H12	15.0% + 1%	0.6	734.8	367.40	34.56

* highest unconfined compressive strength, the best mixture; ** Curing conditions: 100% humidity, 25 °C, 28-day.

**Table 3:** Strain–ductility ratio of stabilized quarry fines at 7-day and 28-day ages.

Type of Specimen	Cement-Stabilized Quarry Fines		Ecolan-Stabilized Quarry Fines		Fly Ash-Stabilized Quarry Fines	
Curing Days	7-day	28-day	7-day	28-day	7-day	28-day
Average SDR	3.64	3.63	2.14	2.24	1.95	2.45
* $\varepsilon_{y,ave}$	0.00817	0.00829	0.0188	0.0164	0.0153	0.0108
** $\varepsilon_{f,ave}$	0.0144	0.0139	0.0234	0.0237	0.0175	0.0148

* Average first yield strain; ** Average strain at failure.

**Table 4:** Indicators to evaluate effects of freeze-thaw cycles.

Type of Specimen	Cement-Stabilized Quarry Fines		Ecolan-Stabilized Quarry Fines		Fly Ash-Stabilized Quarry Fines	
Freezing-Thawing Conditions	No Freezing-Thawing Cycles	10 Freezing-Thawing Cycles	No Freezing-Thawing Cycles	10 Freezing-Thawing Cycles	No Freezing-Thawing Cycles	10 Freezing-Thawing Cycles
Average SDR	1.81	1.50	1.76	1.23	1.41	1.36
$\varepsilon_{y,ave}$	0.0149	0.0201	0.0231	0.0332	0.0169	0.0190
$\varepsilon_{f,ave}$	0.0204	0.0240	0.0308	0.0379	0.0193	0.0211
FTR	0.946		0.808		0.662	
